# Editorial: High-Intensity Exercise in Hypoxia: Beneficial Aspects and Potential Drawbacks

**DOI:** 10.3389/fphys.2017.01017

**Published:** 2017-12-04

**Authors:** Grégoire P. Millet, Olivier Girard

**Affiliations:** ^1^Faculty of Biology and Medicine, Institute of Sport Sciences, University of Lausanne, Lausanne, Switzerland; ^2^Athlete Health and Performance Research Centre, Qatar Orthopaedic and Sports Medicine Hospital, Doha, Qatar

**Keywords:** altitude training, deoxygenation, repeated sprint training in hypoxia, resistance training in hypoxia, muscle activation, HIF-1α, ischemic preconditioning, hypoxia

## Recent developments in hypoxic training

With the recent development of new altitude training methods (Millet et al., 2013; Girard et al., 2017), the question of the specific central and peripheral adaptations to high-intensity exercise in hypoxia is now crucial. This research topic investigated the beneficial aspects and potential drawbacks of these methods and would undoubtedly be of interest for many exercise physiologists. A total of 16 papers have been accepted, arising from 18 different research groups from 12 countries. Four different main areas have been investigated:

1. High-intensity, continuous exercise in hypoxia.
2. Repeated sprint training in hypoxia.
3. Resistance training in hypoxia.
4. Therapeutic use of hypoxia.

## High-intensity, continuous exercise in hypoxia

Van Thienen et al. investigated the HIF-1 pathway (from vastus lateralis biopsies) in 11 monozygotic twin pairs who performed an experimental trial in both normoxia and hypoxia. They tested the hypothesis that this pathway and its downstream targets in energy metabolism are regulated in a genotype-dependent manner. A key observation was that hypoxic exercise-induced increment of muscle HIF-1a mRNA content was about 10-fold more similar within monozygotic twins than between the twins. Authors concluded that genetic factors play an important role in the muscular responses to acute hypoxic stress at rest and during exercise and that the regulation of HIF-1a stabilization in acute hypoxia is genotype-dependent.

Townsend et al. computed the critical power (CP) and the work above CP (W') in male cyclists performing time trials in normoxia and at five different altitudes from 250 to 4,250 m. They predicted performance during a high-intensity intermittent test performed in normoxia and in normobaric hypoxia (simulated altitude: 2,250 m). They reported a curvilinear decrease in CP with increase in altitude severity as well as a significant decrease in W′ occurring only at 4,250 m. Practically, this study enables the prescription of equivalent relative intensity interval training workouts in hypoxic conditions compared with normoxia.

Black et al. explored whether there is a difference in the percentage of VO2max achieved (during a 2-min exhaustive run) in normoxia and hypoxia in 14 middle distance runners. Compared to normoxia, VO2max was lower during a ramp test and VO2 kinetics (greater time constant of the primary phase) were slower in hypoxia. Whereas the runners were unable to reach VO2max during the exhaustive constant work-rate run lasting ~2 min in normoxia, they were able to achieve the reduced VO2max in a hypoxia despite slower VO2 kinetics.

Torres-Peralta et al. investigated the contribution of central and peripheral mechanisms during exercise to exhaustion in normoxia and hypoxia. Following the exercise to exhaustion, legs circulation was occluded during 10 or 60s for impeding recovery and increasing the metaboreflex. The fact that task failure was apparently not due to muscle peripheral fatigue, but instead primarily resulted from reduction in muscle activation, highlights the importance of central mechanisms.

The same research group Torres-Peralta et al. investigated the role played by different levels of inspired pressure in O2 (P_I_O_2_) on muscle activation during exhaustive exercise. A unique observation was that the increase in P_I_O_2_ at exhaustion reduced fatigue and allowed exercise continuation. This study therefore indicates that severe hypoxia induces larger central fatigue (decrease in muscle activation).

## Repeated sprint training in hypoxia

Decrease in convective factors, in turn leading to a reduced training intensity (i.e., not sufficiently intense to stress O_2_ delivery and maximized adaptations), is an inherent characteristic of interval-training in hypoxia (IHT) compared with normoxia. To overcome this limitation, the so-called “repeated-sprint training in hypoxia” or RSH has been developed as a new intervention in our laboratory in Lausanne (Faiss et al., 2013a): with exercise intensity being maximal during RSH we have postulated that this would allow a better recruitment of fast-twitch muscle fibers (Faiss et al., 2013a,b). An up-regulation of circulating microRNAs levels was observed only when exercise was performed at high-intensity and high altitude (i.e., and not at lower intensity) and therefore RSH training is based on the repetition of short (<30 s) “all-out” sprints with incomplete recoveries in hypoxia (Vogt et al., 2001; Faiss et al., 2013a). Hence, a lower rate of O_2_ delivery to the muscles increases the stress on glycolytic flux, which may stimulate the up-regulation of this energy pathway. Compared with repeated-sprint training in normoxia (RSN), RSH could induce beneficial adaptations at the muscular level, along with improved blood perfusion, which may lead to greater improvements in repeated-sprint ability.

Superior repeated-sprint ability in normoxic conditions has been associated with completion of RSH vs. RSN in cohorts of rugby players (Galvin et al., 2013), well-trained cyclists (Faiss et al., 2013b), cross-country skiers (Faiss et al., 2015), soccer players (Gatterer et al., 2014; Brocherie et al., 2015a), field hockey players (Brocherie et al., 2015b). The effectiveness of RSH was confirmed by a recent meta-analysis based on 9 controlled studies (all published in the past 4 years) showing larger mean performance (Brocherie et al., 2017).

Blood lactate accumulation is higher at a simulated altitude of 4,000 m when compared with more moderate simulated altitudes during repeated treadmill sprints (Goods et al., 2014). In an opinion letter, Scott et al. stated that RSH led to a greater reliance on anaerobic metabolism. This increased metabolic stress is likely to promote peripheral fatigue resistance induced by RSH.

Using near-infrared spectroscopy it was previously reported that prefrontal cortex, but not muscle, oxygenation is impaired when ten, 10-s sprints (with 10 s of rest) are completed at 13 vs. 21% oxygen (Smith and Billaut, 2010). For the first time, Willis et al. compared muscle and cerebral oxygenation trends during a one-off RSH trial performed to exhaustion in normoxia (400 m) and at two different simulated altitudes (2,000 and 3,800 m). There was a continual decrease in convective factors of oxygen delivery (e.g., decreases in pulse oxygen saturation and peak oxygen uptake) with increased hypoxia severity, which was linked with impairment in performance (number of sprints and total work) across conditions. Cerebral deoxygenation demonstrated greater changes at 3,800 m compared with 400 and 2,000 m, as well As well as larger deoxygenation/reoxygenation levels during sprints/recoveries near exhaustion. These results show that central autoregulation (i.e., increase in cerebral perfusion near exhaustion) occurs in order to continue exercise despite limited peripheral and cerebral oxygen delivery, until a certain point of limited diffusion at which protective mechanisms cause exercise cessation.

Sweeting et al. reported that repeated-sprint and single-sprint efforts are compromised at 3,000 m simulated altitude, possibly due to limited muscle O_2_ availability during recovery periods. Whilst repeated-sprint and single-sprint efforts were maintained at 2,000 m, the elevated physiological demands at 3,000 m may have been overwhelming.

Girard et al. investigated the neuromuscular adjustments following repeated treadmill sprints at simulated altitudes of 1,800 and 3,600 m or in normoxia. Post-exercise decrease in voluntary strength of knee extensors was greater at 3,800 m than at 1,800 m and normoxia. However, the exercise-induced alterations in rapid torque development were similar between the three conditions.

De Smet et al. investigated if oral nitrate intake influenced buffering capacity and fiber type distribution (vastus lateralis biopsies) after 5 weeks of sprint interval training in hypoxia or in normoxia. Altogether, sprint interval training in hypoxia did not lead to enhanced aerobic or anaerobic endurance exercise performance but oral nitrate supplementation increased the proportion of type IIa muscle fibers. Richardson et al. reported that the improvement in VO_2peak_ and the inflammatory responses (IL-6 and TNFa) were similar after 2 weeks of repeated sprint performed in normoxia or in hypoxia. However, improvement in anaerobic threshold was observed only after RSH.

Hamlin et al. reported the performance changes after six sessions of RSH vs RSN in 19 well-trained male rugby players. These authors confirmed the effectiveness of RSH since repeated sprint performance was enhanced to a larger extent whereas the aerobic performance did not change.

## Resistance training in hypoxia

There is growing research interest focusing on the so-called “resistance training in hypoxia” (RTH) (Scott et al., 2014). A classical reasoning is that, in an O_2_-deprived environment, the low partial pressure of O_2_ would increase metabolite (e.g., blood [La] and anabolic hormones [e.g., growth hormone]) accumulation, leading to an accelerated recruitment of higher threshold motor units (Manimmanakorn et al., 2013) and a subsequent higher hypertrophy with eventually greater improvements in muscle strength (Scott et al., 2015).

Here, Inness et al. examined the effects of 20 sessions of heavy resistance training performed either in hypoxia (RTH) or in normoxia. RTH induced a larger enhancement in absolute and relative strength as well as 1RM.

Paradis-Deschênes et al. investigated the effects of ischemic preconditioning on knee extensions in strength-trained male vs. female athletes. Males reported a greater peripheral oxygen extraction and greater strength enhancement than females.

That said, a recent meta-analysis (Ramos-Campo et al., 2017) concluded that RTH did not provide significant benefit for muscle size and strength over resistance training in normoxia. This highlights the need for additional research on this burgeoning area.

## Therapeutic use of hypoxia

Potential benefits of using hypoxia exposure therapeutically have been recently suggested for elderly, obese or hypertensive patients (Millet et al., 2016).

Here, Girard et al. postulated that hypoxic walking would be beneficial in obese patients since it might lead to a decreased walking speed and subsequently a lower biomechanical load.

Along the same lines, Pramsohler et al. assessed the physical effort in geriatric patients (age > 65 years) for the same HR response in normoxia or in hypoxia (simulated altitude: 3,000 m). The main benefits of the hypoxic sessions were a lower stress on the locomotor systems for a similar physiological strain than in normoxia.

## Conclusion

As confirmed by this research topic, high-intensity exercise in hypoxia is a growing area of interest. Ergogenic effect? Stamped! Adaptive mechanisms? More investigation needed! Therapeutic usefulness? The future!

## Author contributions

All authors listed have made a substantial, direct and intellectual contribution to the work, and approved it for publication.

### Conflict of interest statement

The authors declare that the research was conducted in the absence of any commercial or financial relationships that could be construed as a potential conflict of interest.
